# From Bureaucratic Coordination to a Data-Driven Model: Transformation and Capacity Building of Community-Based Prevention and Control of Public Health Events

**DOI:** 10.3390/ijerph19148238

**Published:** 2022-07-06

**Authors:** Mingxia Xian, Chong Zhao, Yicheng Zhou

**Affiliations:** 1 School of Political Science and Public Administration, Excellent Innovation Team of Local Government and Social Governance, Soochow University, Suzhou 215123, China; mingxiaxian2022@163.com; 2 School of Marxism, Nanjing University of Information Science and Technology, Nanjing 210044, China; zhaomarcia@163.com; 3 Center for Chinese Urbanization Studies, Soochow University, Suzhou 215123, China; 4 Collaborative Innovation Center for New Urbanization and Social Governance, Soochow University, Suzhou 215123, China

**Keywords:** bureaucratic coordination, data driven, public health events, community-based prevention and control capacity

## Abstract

Communities are the first line of defense in responding to major public health events. Taking the community-based prevention and control cases of COVID-19 in China as samples, this paper constructs an analytical framework for the generation of community-based prevention and control capacity of public health events from the perspective of governance elements optimization based on the methods of text analysis and limits-to-growth archetype analysis. According to the research, the community-based prevention and control of public health events realizes the integration of governance elements of key actors through the bureaucratic coordination mode and maximizes the prevention and control efficiency with the primary goal of epidemic prevention and control in a short period of time, which presents a “reinforcing feedback” loop in the “limits-to-growth” model system. However, with the development of the epidemic showing a strong trend of being latent and wide spread, the “reinforcing feedback” from the bureaucratic coordination model on the effect of epidemic prevention and control encounters the “regulatory feedback” that inhibits the growth at the data-driven level. On the basis of discussing the practice of the public health prevention and control mode in the grassroots communities under the established political framework, this paper attempts to construct an institutional reform system from technological governance to technological empowerment, so as to effectively realize the mode transformation of community-based prevention and control of public health events.

## 1. Introduction

As the most widespread and most serious global public health emergency in modern times, COVID-19 has had a huge impact on global economic and social development and reshaped international relations and national development to varying degrees. At the same time, it also creates a natural experimental research scenario for policy researchers to observe the impact of public health events on grass-roots governance capacity and emergency prevention and control capacity. In the context of the COVID-19 pandemic, this public health crisis has affected all walks of life in the whole region, and the community has provided an indispensable organizational level and prevention and control barriers for the participation of the whole region [1]. In March 2020, when General Secretary Xi Jinping inspected the epidemic prevention and control work in Wuhan, he pointed out that China’s epidemic prevention and control mainly includes two main fronts: hospitals and communities [2]. As the front line of the prevention and control of major public health events, communities have played an important role in the prevention and control of public health events in China and have formed a “community”-based paradigm for the prevention and control of public health events in China. From the perspective of practical incrementalism, the epidemic prevention and control strategy is a political calculation based on economic growth under capital accumulation and social stability under government legitimacy [3]. In the federal system where the health care power is divided between the federal and state governments, there are different degrees of conflict in the order of providing, paying and distributing public health resources, which are political processes that have an important impact on public health. For example, the anarchy pursued by Brazil in response to the COVID-19 pandemic can be called a disaster in the history of public health prevention and control [4]. Different from the national response strategy under the logic of individual liberalism, China’s epidemic prevention and control adopts a “nationwide system” based on strong resource allocation capacity, full mobilization and control capacity. On the one hand, China’s public health and community fields have not established a relatively close relationship, which is different from the relatively mature experience and practice of social work in the field of public health in developed countries, such as the United States. On the other hand, due to the shortcomings of the existing early warning mechanism, epidemic prevention system and hierarchical diagnosis and treatment system in China’s public health field, community-based public health prevention and control is not embedded in the existing public health system, but embedded in the administrative system, such as community work.

The prevention and treatment of public health emergencies is no longer a simple medical technology issue, but a complex global governance issue [5]. Starting from the case of COVID-19 prevention and control in China, it is of great practical significance to explore the realization process of community-based prevention and control of public health empowered by big data under the guidance of public institutions and clarify the basic characteristics and governance path of public health prevention and control mode of grassroots communities under the established political framework. In the abnormal stage of epidemic prevention and control, the community-based prevention and control of public health events realizes the integration of governance elements for key actors through the bureaucratic coordination model and maximizes the prevention and control efficiency with the primary goal of epidemic prevention and control in a short period of time. However, in the normal stage of epidemic prevention and control, this is highly unsustainable in terms of cost and scale, resulting in the lack of capacity of the bureaucratic coordination model. Based on the “limits-to-growth” archetype analysis of the constraints on the improvement of community-based prevention and the control capacity of public health events, an analytical framework for the generation of community-based prevention and control capacity of public health events from the perspective of optimization of governance elements is formed to effectively explore the governance system and governance capacity empowerment mechanism of community public health prevention and control.

## 2. Literature Review

The challenges posed by the COVID-19 pandemic have gone beyond public health and are inextricably linked to global governance and public policy [6]. As a country with a large governance scale, coupled with the suddenness and variability of risk prevention and control of public health emergencies, it is of great practical significance for China to strengthen the prevention and control of public health emergencies at the community level. Existing studies on governance innovation mainly focus on the development and reflection of governance theory, the evolution and development of governance types, and the effectiveness and exploration of governance practices [7], while research on the optimization of governance elements and the construction of governance capacity at community level is relatively scarce. Given that, it is necessary to conduct a preliminary discussion on the community turn of public health event prevention and control and the optimization of governance elements under the two prevention and control models of bureaucratic coordination and the data-driven model.

### 2.1. Community Turn of Public Health Event Prevention and Control

The so-called community is a living community with stable physical boundaries and common value orientation. Compared with the more common definition of the community as a physical carrier, the community has gradually become the dominant tool to deal with public health emergencies in methodology. On the one hand, from the perspective of the physical attributes of the community, as the most normal living place for ordinary people, the community carries the temporary response to the current sudden public crisis. On the other hand, from the perspective of the social attributes of the community, the recessive resources within the community, such as the trust capital within the community, have played an assisting and supplementary role in the recovery of mental health after a public health crisis to a certain extent.

The COVID-19 pandemic, the rampant spread of mutant strains of the virus and the protracted epidemic prevention and control actions indicate that the community prevention and control mode of public health events has shifted from temporary intervention to long-term intervention, which also provides an opportunity for the community turn of prevention and control of public health crises [1]. In the field of public health prevention and control, countries such as the United Kingdom and the United States have established relatively mature community-based public health medical treatment systems and facilitated cultural communication and have generally established community-centered public health participatory research strategies [8]. In terms of community public health research, western countries divide the population of community residents to study the relationship between the political and economic status of different residents and the level of public health. For example, the possible associations between socioeconomic, cultural, lifestyle, genetics, susceptibility, etc., and the pathophysiological manifestations are explored through the data of COVID-19 and previous pandemic infections in the BAME community (Black, Asian and minority ethnic communities) [9]. Some scholars have explored the public health prevention and control measures of religious groups through the practice progress of religious communities in carrying out basic public health work [10]. In the field of Chinese governance, the community is not only a living community in the sense of a physical carrier, but also a community department in an administrative sense. However, whether it is the outer edge of any of the above two concepts, the community turn in the prevention and control of public health emergencies is characterized by temporary and transient characteristics to a certain extent. This responsive governance model, which focuses on response and recovery, is characterized by a permanent lag in action over the course of the epidemic. Risk communication and community participation are the core parts of a public health emergency response [11], so how to change the community emergency response from a short-term and one-way communication to normal and interactive community habits so as to form a sustainable community prevention and control ability of public health events is the focus of this paper.

### 2.2. Governance Logic of Community-Based Prevention and Control of Public Health Emergencies

Existing research has summarized two governance logics to deal with the uncertainty of crisis events—decision logic and creation logic. The former presents a top-down responsive governance model. By responding to the social governance needs of crisis events as the core, it is essentially taking corresponding remedial and disposal measures for social crisis problems that have occurred through the bureaucratic coordination mechanism. The latter emphasizes bottom-up technological innovation and systematic application and reduces the uncertainty and harm of risks through crisis early warning. However, if these two governance logics only have a one-dimensional governance tendency, they will present strong governance dilemma and operation limit when facing the public health emergencies with large scale, long duration and wide harm. From the perspective of governance elements, this study refines the governance path of community-based public health event prevention and control through the integration and optimization of risk prevention and control elements.

#### 2.2.1. Bureaucratic Coordination Model under the Decision Logic

As an important concept in rational bureaucracy, bureaucratic coordination usually refers to a vertical coordination mechanism based on legal authority and institutionalized rules. Weber once said, “The typical legal ruler is the ‘superior’… His orders are system-oriented… Any institution has a fixed system of supervision and inspection” [12]. Stillman II pointed out that bureaucratic coordination is “the separation of superiors and subordinates, and the distribution of wages, authority, privileges and promotions in this hierarchical order… Bureaucrats cannot act arbitrarily, their options are subject to statutory codes of conduct and limitations” [13]. As an organizational coordination model that is compatible with industrial society and coordinated with market organization and social organization, bureaucratic coordination emphasizes vertical centralized management, hierarchical communication and coordination and impersonal management, and it has been considered as one of the most effective organizational coordination models.

In the process of prevention and control of public health events, the bureaucratic coordination in the context of Chinese government governance mainly adopts “pressure-based system + campaign-styled governance” to realize the optimization of governance elements. Pressure-based system refers to a system model in which lower-level political organizations operate under the pressure of the evaluation system and various indicators formulated by higher-level political organizations. In order to complete the tasks and indicators set by higher-level political organizations, political organizations at all levels will quantify and decompose tasks and indicators and push them down to lower-level political organizations and individuals. Meanwhile, the lower-level political organizations will be assessed, rewarded and punished according to the completion of tasks [14]. The campaign-styled governance mechanism replaces the conventional process with political mobilization, adopts the method of public opinion build-up and comprehensive mobilization to stop the inertia and rhythm of the conventional mechanism, transmits the intentions and signals of the central government to various departments and fields in a short period of time, rectifies “deviations” and standardizes boundaries in the political governance of local governments at all levels so as to avoid the organizational failure of conventional mechanisms [15]. The pressure-based system and the campaign-styled governance mechanism interact and promote each other, so that the bureaucratic coordination governance model shows the institutional advantages, such as the optimal allocation of various governance elements and the efficient completion of governance tasks in the process of public health event prevention and control. Among them, the pressure-based system efficiently mobilizes the enthusiasm, initiative and creativity of lower-level organizations and individuals through a “pressure-driven” model and ensures the completion of tasks and indicators by uniformly allocating people, finance, materials and other governance elements. Campaign-styled governance derives its legitimacy and organizational basis from national movements, which attempts to break down institutional, conventional and professional boundaries and mobilizes needed social resources through strong state mobilization. Campaign-style governance can “mobilize all positive factors” in the optimization of governance elements in the short term to achieve the goal of “concentrating efforts to do major things” [16]. In the process of bureaucratic coordination in the prevention and the control of public health events, campaign-style governance is often embedded in a pressure-based system in order to mobilize various governance elements quickly and efficiently.

However, the bureaucratic coordination governance model can only show high efficiency in a specific applicable field. Williamson pointed out that there are two limitations for bureaucracy to break through the framework of organizational failure: one is to withstand scale and transaction constraints; the other is to follow the appropriate principles of decomposition hierarchy and provide necessary incentives and control means [17]. Beetham, Ostrom and others believe that there are asymmetric problems, such as information overload, information shortage and resource load in bureaucratic coordination, and the effective implementation of its mechanism should be based on the assumption that the government has sufficient information resources, strong supervision and zero expenditure of administrative expenses [18]. In addition, the pressure-based system and the campaign-style governance mechanism also have limitations in the integration of governance elements. The pressure-type system can easily lead to the contradiction between fiscal restrictions and the expansion of local government functions, as well as the rent-seeking problem of “official entrepreneurs” [19]. Campaign-style governance is a rational decision of the government under the condition of poor and weak governance resources. It can quickly optimize the allocation of governance elements in a short period, but it is difficult to normalize the optimal allocation of governance resources [20]. The bureaucratic coordination under the bureaucratic structure is prone to “friction between the policy and the bureaucratic organization, which reduces the implementation of the policy, and results in the abolition of the policy or the distortion of the implementation” [21] in the implementation of national policies.

#### 2.2.2. Data-Driven Model under the Creation Logic

Technology empowerment innovation based on creation logic is forming a set of application-based social problem-solving mechanisms [22]. The data-driven model not only includes the acquisition of massive heterogeneous data but also the process of discovering, analyzing and extracting data value to support decision making [23]. The data-driven model based on big data is the core mechanism of governance in the digital era. The value of big data [24] transcends data itself and combines with specific process conventions to generate driving value [25]. As a disruptive technological change in the 21st century, the process and method of data-driven management and decision-making research are being redefined. Big data-oriented management decisions will clarify the interactivity of massive and complex data to reduce the information asymmetry caused by data redundancy or lack of data and carry out collaborative management with the flow and utilization of data among multiple subjects. In general, the data-driven model has great potential to advance the optimization of governance elements, and “Data will not be consumed by the ideas and innovations it inspires, on the contrary, it can provide endless fuel for innovation…… The power of the data will be magnified because of these innovative ideas” [26].

To a certain extent, public health events, such as the COVID-19 outbreak, have become accelerators for governance and related changes in the digital age [27]. In the governance of digital age, “data driven” is defined as a data-based management decision-making model [28]. On the one hand, the data-driven model promotes the optimization of governance elements, which in turn promotes the synergy of governance functions. By building a data resource exchange platform, establishing “one-network” services, breaking down “data barriers” and “information islands”, the cross-regional, cross-department and cross-level information sharing and coordination movement of big data resources can be fully promoted, the service process can be optimized, and multi-subject co-governance can be realized [29]. In emergency management, data-driven governance can solve the dilemma of the “data capability gap” in bureaucratic coordination, realize “reconstruction of data capabilities”, break the professional division of bureaucratic coordination through data flow and enhance multi-department coordination in emergency response. On the other hand, data-driven construction of intelligent services improves the scientific and democratic degree of decision making and realizes the precision and intelligence of the governance process in “technical governance”. For example, by using the data technology that captures the management object, it can track and analyze its unique needs so as to realize the refinement and personalization of emergency management [30].

### 2.3. Research Review

The bureaucratic coordination model under the logic of political decision making provides an institutional environment for condensing the interests of multiple parties in a short period of time, and the technological drive under the logic of creation provides internal motivation and expansion for the improvement of risk governance efficiency [31]. Qu Jingdong believes that the development process of the modernization of the national governance system and governance capacity is the development process of the problem of technology adapting to production and life under the coordination of bureaucracy [32]. In discussing the realization mechanism of big data on public health risk management, scholars took China’s COVID-19 prevention and control as a case study and believed that the data-driven realization path should be deeply integrated with technological innovation and management optimization [33]. The opening and sharing of data, with the participation of multiple subjects and the public is a process of “ technical empowerment “ for community-based prevention and control of public health events, which helps community governance step into a win-win era of interaction between the public and data.

Existing studies mainly focus on the evaluation of community-based prevention and control capacity, emergency response effect [34] and the establishment of the index system of community-based prevention and control capacity of public health events [35], such as the construction of urban community epidemic prevention and control information governance system, but ignores the research on the generation process of community-based prevention and control capacity of public health events in different prevention and control models. Although existing studies on community-based prevention and control capacity of public health events have focused on the role of key elements in emergency management on capacity building, for example, some scholars have analyzed early warning management behaviors and key factors in major public health emergencies, revealing the building elements of early warning management capacity [36], and some scholars have proposed to establish community solutions related to the driving factors affecting healthy society [37], but studies rarely analyze the generation process and internal mechanism of community-based prevention and control capacity of public health events from specific governance fields, and mainly focus on the static analysis of the community-based prevention and control capacity building of public health events, which is difficult to reveal the internal law of capacity building.

## 3. Research Methods and Analytical Framework

### 3.1. Research Methods

Although the COVID-19 crisis has caused extensive and in-depth impacts in different policy areas of countries around the world, it is not yet a paradigm shift in the traditional sense of a critical juncture [38]. Technology-driven governance reflects the basic orientation of adaptive transformation of social governance [39]. On the one hand, as a general tool, although the implementation and introduction of big data accelerates the informatization of governance methods and processes to a certain extent and promotes the realization of organizational goals, it does not change the operational logic of the bureaucratic system, or even solidify the original organizational structure. On the other hand, the institution enters the institutional implementation framework through the form of cognition, culture, norm and social structure embeddedness. In summary, the bureaucratic coordination model and the technology-driven coordination model build an institutional reform system from technological governance to technological empowerment through the two-way embedding of decision logic and creation logic and effectively realize the model transformation of community-based prevention and control of public health events.

In terms of method selection, the research mainly adopts the methods of text analysis and limits-to-growth archetype analysis. By extracting information from the cases of community-based prevention and control capacity building, and in the stage of integration and refinement of coding-based conceptual categories, government officials and relevant experts are invited to test and demonstrate the axis codes one by one to ensure the objectivity of the results. At the same time, the limits-to-growth model of the constraints on the improvement of community-based prevention and control capacity of public health events is constructed, and an analytical framework for the generation of community-based prevention and control capacity of public health events from the perspective of optimization of governance elements is formed.

### 3.2. Analytical Framework Construction

After research data coding and expert opinion consultation, the influencing factors of community-based prevention and control capacity construction of public health events are determined as four dimensions of “information-resources-subject-mechanism”. First, information is the driving element of governance innovation and community prevention and control capacity building. Data-driven governance applies to information governance principles, including organization, metadata, privacy, data quality, business process integration and information life-cycle management [40]. The essence of the data-driven model is to endow the connotation of information to the data as a symbol or carrier for effective communication and management. Data integration and information sharing can only be realized when massive big data are presented as regular information through knowledge and flows between governance subjects and governance platforms. The difference between the bureaucratic coordination model and the data-driven model lies in the degree of integration of basic information, which results in different driving forces for later prevention and control capacity. Second, resources are the fundamental element for governance innovation and community-based prevention and control capacity building. The building of community-based prevention and control capacity of public health events requires the full mobilization of human and material resources inside and outside the community. The model of the bureaucratic coordination of prevention and control adopts the method of political mobilization to mobilize the party members and cadres of the grass-roots government vertically to the community and coordinates the resources in the system; the data-driven prevention and control model coordinates the forces of all parties horizontally through the sharing of service data so as to coordinate all resources. Third, the main body is the initiative element of governance innovation and community-based prevention and control capacity building. The building of community-based prevention and control capacity of public health events involves multiple governance subjects, such as grass-roots government, community party organizations, residential self-governance organization, community property management organizations, owners committee, enterprises inside and outside the community, social organizations and community residents. Through governance innovation, fully mobilizing the enthusiasm of these governance subjects and forming a positive interactive relationship between governance subjects are key to improving the community’s ability to prevent and control public health events. Fourth, the mechanism is an integral element of governance innovation and community-based prevention and control capacity building. Mechanism linkage can effectively break down information barriers and data sharing barriers between horizontal departments and governance subjects in bureaucratic coordination, remove institutional barriers in resource sharing and realize information opening and sharing and multi-subject cooperation and co-governance among multiple bodies, which is an important guarantee for the improvement of community-based public health prevention and control capacity.

**Figure 1:**
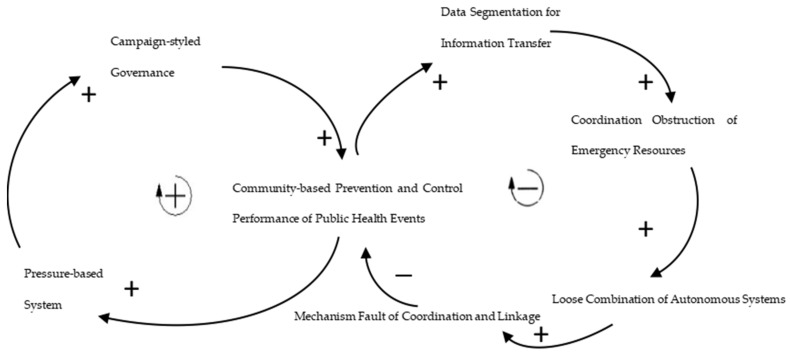
Analytical framework: “Limits-to-growth” archetype of the constraints on the improvement of community-based prevention and control capacity of public health events. Source: self-made.

The above-mentioned factors influencing the improvement of community-based prevention and control capacity are not isolated but interact and restrict each other, forming a dynamic and complex system. Based on the feedback model in system dynamics, this study attempts to establish the limits-to-growth archetype of the constraints on the improvement of community-based prevention and control capacity of public health events and form an analytical framework for the generation of community-based prevention and the control capacity of public health events from the perspective of optimization of governance elements (see Figure 1).

The community-based prevention and control system for public health emergencies is characterized by multiple feedback loops and hysteresis effects of system dynamics. The “limits-to-growth” archetype system mainly includes the “reinforcing feedback” of the bureaucratic coordination model under the decision logic and the “regulatory feedback” of the technology-driven model under the creation logic. Taking the COVID-19 outbreak in 2020 as an example, China adopted extensive political mobilization in epidemic prevention and control and realized an efficient response in the initial stage of epidemic prevention and control through vertical centralized management, which fully reflected the institutional advantages of Chinese governance with Chinese efficiency. However, with the development of the epidemic showing a strong trend of being latent and wide spread, the “reinforcing feedback” from the bureaucratic coordination model on the effect of epidemic prevention and control encounters the “regulatory feedback” that inhibits the growth at the technology-driven level. The research believes that when there is a limits-to-growth structure in the system, it is useless to promote the “reinforcing feedback” loop, and the reason should be found in the negative feedback loop to eliminate the constraints of the negative feedback loop so as to continuously enhance the positive feedback.

## 4. ”Limits-to-Growth” Analysis of the Constraints on the Improvement of Community-Based Prevention and Control Capacity of Public Health Events

### 4.1. Data Sources

The research adopts a multi-source data collection and multi-case study method. This involves collecting and sorting out 425 pieces of relevant information, such as government announcements, work summaries, research reports, news media reports, mainstream website reports and reprints related to community anti-epidemic. According to the three types of community epidemic prevention and control implementation bodies, government departments, grass-roots communities, enterprises and institutions and social organizations, 38 cases of community-based prevention and control capacity building were sorted out. Through content coding, data mining and in-depth analysis of these multi-source materials, the key concepts and content representations of the “capacity shortcoming” and “technological empowerment” of community-based public health prevention and control are explained. (Some examples of case coding are shown in Table 1).

### 4.2. Practical Dilemma of Community-Based Prevention and Control of Public Health Events under the Bureaucratic Coordination and Governance

In the early stage of public health events, community-based prevention and control mainly adopted the model of bureaucratic coordination. This model has quickly contained the spread of public health events, reflecting the institutional advantages and high efficiency of governance in China. However, it also has a capacity shortcoming in community-based prevention and control, which needs to realize the adaptive transformation of the community-based prevention and control model of public health events. In the context of the Chinese system, the function of government governance is mainly based on the ability of bureaucratic organization and coordination, while the bureaucratic coordination in the prevention and control of public health events has the tension characteristic of “complex”. On the one hand, it presents a pressure-type hierarchical relationship in the structure, with the characteristics of efficient administration [41]. The prevention and control of public health events reflects the return of the government in social governance, and the inward turning trend has occurred in all countries in the world, which also responds to the “inward-looking” stage of the government in the prevention and control of public health events under the bureaucratic coordination. On the other hand, bureaucratic coordination also has the inherent “counter-function” problem of the bureaucratic system [42], which is mainly manifested in the data segmentation of information transmission in the process of community-based prevention and control of public health events, the coordination obstruction of emergency resources under limited human and financial resources and multiple prevention and control functions, the loose combination of autonomous system at the main body level and the mechanism fault of coordination and linkage at the mechanism level.

#### 4.2.1. Data Segmentation of Information Transmission

Although the community is an autonomous mass organization, it belongs to the end of the extension of the bureaucratic boundary under the bureaucratic coordination model. In practical work, it is subject to multiple managements, such as the streets and the cities and counties to which it belongs, and the functions and work boundaries are blurred. The information construction platforms of various departments, such as health, transportation, safety, labor unions and even communities, are different, and community prevention and control of public health events presents a segmented digitization of information transmission.

First of all, in terms of the carrier of information transmission, the non-community subjects have mastered the advantages of technology and platform are profit-driven and take economic benefits as the main goal, which results in repeated construction, lack of functions and low resource integration of the community information resource platform and makes it difficult to form governance synergy under the closed-loop of prevention and control. Secondly, from the in-depth integration of information transmission, a large amount of qualitative information and quantitative data are accumulated and formed in the community-based prevention and control of public health events within and between communities. Under the barriers of multiple and complex data, there is a lack of data association and information integration between massive data and information, resulting in a lack of overall understanding of the latest developments of public health events in the community, and a long time lag between the implementation of prevention and control measures and the current situation of community-based prevention and control. Finally, from the perspective of flexible coordination of information transmission, the peacetime and wartime coordination of community-based prevention and control of public health events is lacking. The current prevention and control measures are mainly policy deployments at national and regional levels based on the judgment of the overall situation, and there is a lack of flexible management and control combined with relevant information, such as community risk levels and the development and changes of public health events.

#### 4.2.2. Coordination Obstruction of Emergency Resources

As with public crisis emergency management events, public health events have imposed strict requirements and severe tests on the coordination and response speed of emergency resources, as well as the resources and capacity of public health treatment. However, in the face of significant daily management work, the community has formed the appropriate community management model to a certain extent under the long-term exploration and consultation. However, this management model is an institutionalized informal behavior, resisting the emergence of certain focal issues or recovering losses [43], which is more common in the current practice of community response to public health events.

By analyzing the governance system of community prevention and control of public health events, it is not difficult to find that community-based prevention and control of public health events mainly includes community-based prevention and control management functions for both peacetime and wartime, such as information mapping, prevention and control monitoring, key population management, access management, information reporting, publicity and education, environmental renovation, difficult support and community services. The community not only performs the management and coordination work in times of crisis, but also connects the medical system with the disease management work. However, the current community construction work based on community prevention and control resources and community health service work based on community rescue resources lack organic linkage. Under the environmental conditions of a public health event outbreak, the adaptive community management model is faced with multiple challenges. First, communities are assuming more and more functions of social governance, making it difficult to maintain coordination of prevention and control resources. The performance of the multiple functions of community-based prevention and control of public health events depends on the limited number of community workers to implement them with their dedication and initiative. In practice, many communities are faced with problems, such as insufficient prevention and control manpower, shortage of prevention and control materials and poor prevention and control conditions. Secondly, in terms of coordination of public health treatment resources, the existing treatment system in the community is overwhelmed. The limited emergency prevention and control resources, equipment and diagnostic capacity of community hospitals and public health service centers lead to the dilemma of “resource allocation failure” in community hospitals and public health service centers in the prevention and control of public health events. Finally, the current allocation system of community emergency resources is mainly the strip-based configuration system, which lacks a realistic investigation of the actual needs of each community and the community’s own coping capacity, which leads to a lack of pertinence and the long-term effect of resource allocation.

#### 4.2.3. Loose Combination of Autonomous Systems

The adaptation of community to the environment can be divided into mechanical adaptation and organic adaptation. The mechanical adaptation is compatible with a stable external environment; while emergency prevention and control are faced with an unstable external environment, so an organic organization with decentralized decision-making power should be established [44]. However, under the realistic background of intensified vertical administrative mobilization and insufficient horizontal social mobilization, the bureaucratic coordination formed a mechanical task distribution system with street as the guidance, community workers as the dominant and the participation of community health institution personnel, community police, volunteers, social organizations and other multi-subjects. In the bureaucratic coordination model, the community has completed the construction of an internal multi-coordinated organizational system, that is, a core circle multi-coordinated structure composed of party cadres, community cadres and staff, property companies, community committees, community police and community doctors.

The loose combination of multiple subjects in community-based prevention and control of public health events brings about the loss of autonomous endogenous order. On the one hand, the community-based prevention and control forces dominated by ordinary residents have not been effectively stimulated and deeply mobilized due to self-social inertia and self-centered selfishness, which is related to the inertia formed by the low participation and the attention of community residents in daily community management. Residents as the main governance body with low participation are prone to the distrust of community prevention and control work and have doubts or even negative attitudes towards the effectiveness or even the motivation of community management, which makes it difficult to effectively form a joint force of grassroots prevention and control in a short time. On the other hand, community prevention and control of public health events presents a weak complex supervision subject relationship under the leadership of the community. Community prevention and control of public health events mainly adopts the community supervision model under grid management. Community police, community medical staff, community organizations, etc., are assigned tasks according to their functions. Each subject represents the role of the group or organization to which it belongs, and lacks community responsibility for community prevention and control, making it difficult to coordinate responses to public health events. Taking social organizations as an example, most of the current social organizations participate in community governance in the form of government purchases. When public health events and other emergencies break out, they lack the experience or ability to actively intervene, and it is difficult to form sustainable governance synergy.

#### 4.2.4. Mechanism Fault of Coordination and Linkage

The problems caused by the loose combination of the community autonomy system are mainly manifested as the mechanism fault of coordination and linkage. Community prevention and control of public health events mainly relies on the exhaustion of community personnel under the “iron footplate”, and a standardized prevention and control mechanism has not yet been formed. Community prevention and control of public health events is mainly manifested in the implementation of the administrative deployment of the higher level and the initiative of the community leaders. There is a multi-subject linkage fault at the horizontal cooperation level, which is mainly manifested in the following two aspects.

On the one hand, in the normalized prevention and control of public health events in the community, the construction of a community-centered emergency prevention and control system is lacking. Under uncertain conditions, such as information vacancy and information overload, community workers generally need to make immediate decisions and implement temporary strategies, such as physical barriers, as the main method when dealing with the prevention and control of unconventional public health events. In addition, the mobilization of stakeholders, such as property management companies, real estate development companies and other market service organizations, group organizations, various volunteer commandos, and enterprises and organizational entities inside and outside the community, is insufficient. In the outbreak process of public health events, although a large number of government resources, personnel and equipment are invested, due to the lack of community emergency management capacity there is a trend of “involution” in the use of these resources.

On the other hand, in the emergency rescue work of public health events, the hierarchical diagnosis and treatment system of community public health prevention and control is faulty. This type of emergency rescue prevention and control fault is manifested in the fact that community hospitals, as grass-roots public health service units, are at the end of the government-led medical organization system of public health event prevention and control based on the bureaucratic system, and the materials, equipment and personnel are still dominated by municipal hospitals at or above the street level. Secondly, the current hierarchical diagnosis and treatment system plays an important role in the care and intervention of chronic diseases, such as diabetes and senile osteoporosis. However, in the prevention and control of major public health emergencies, community hospitals lack normal business cooperation and contact mechanisms in docking with general hospitals, and the bottom-up direct reporting system of public health events is absent. The effectiveness of grass-roots inspection, diagnosis and treatment of community hospitals needs to be further improved.

In general, bureaucratic coordination is a kind of coordination model of prevention and control of public health events through the pressure and mobilization of administrative power, which forms the community-based prevention and control capacity of public health events based on administrative instructions. Under the model of bureaucratic coordination of prevention and control, the grass-roots government plays a leading role in prevention and control, and the community becomes the “administrative end” of the prevention and control of public health events and is in a position of strict compliance with administrative instructions. The bureaucratic coordination model quickly transfers the community-based prevention and control of public health events into a “wartime” state where all staff are deployed, enhances the community’s response to public health event prevention and control, and quickly contains the spread of public health events. However, bureaucratic coordination is a public health event prevention and control model with a high concentration of power and sharp sinking of resources. On the one hand, it suppresses the endogenous self-government ability of the community, and on the other hand, it also has the disadvantage of high cost and unsustainability. After quickly curbing the spread of public health events, the bureaucratic coordination and governance model is facing the development trend of model transformation.

## 5. Analysis of “Technical Empowerment” Strategy for Community-Based Prevention and Control of Public Health Events

### 5.1. Explanatory Model for Data-Driven Community Prevention and Control Capacity of Public Health Events

**Figure 2:**
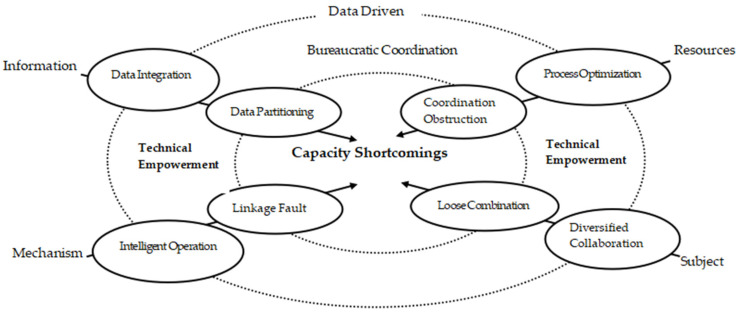
Explanatory model for data-driven community-based prevention and control capacity of public health events. Source: self-made.

The data-driven model is a mode of building community-based prevention and control capacity for public health events for both peacetime and wartime use, which can realize the vertical integration of prevention and control resources, the data closed loop of prevention and control information and the multiple coordination of prevention and control subjects based on digital technologies, such as big data, artificial intelligence, and cloud computing, thereby stimulating the endogenous autonomy of the community and building a digital governance prevention and control model for public health events under the smart community. This model can effectively make up for the “capacity shortcoming” of community prevention and control of public health events under the model of bureaucratic coordination of prevention and control and continuously improve the data-driven community prevention and control capacity of public health events (see Figure 2).

As shown in Figure 2, in the early stage of the bureaucratic coordination model of public health event prevention and control, the community-based prevention and control of public health events has four “capacity shortcomings”: the data segmentation of information transmission, the coordination obstruction of emergency resources, the loose combination of autonomous systems and the mechanism fault of coordination and linkage. In the data-driven model for both peacetime and wartime use after the rapid containment of the spread of public health events, through “technical empowerment”, the data integration of information platforms, the process optimization of resource allocation, the multiple coordination of resource expansion and the intelligent operation of information sharing, a data-driven community-based prevention and control capacity for public health events has been constructed.

### 5.2. Improvement Strategies for Breaking through the “Limits-to-Growth” of Community-Based Prevention and Control Capacity of Public Health Events

From the perspective of practical operation, the process of community-based prevention and control of public health events has gone through two stages: bureaucratic coordination and the data-driven model. In the middle and late stages of public health event prevention and control, the government’s governance strategy for community-based prevention and control usually changes, that is, from government mobilization to social mobilization, relying on big data, artificial intelligence, cloud computing and other digital technologies as technical support. When the model of community-based prevention and control of public health events has changed from bureaucratic coordination to the data-driven model, “technical empowerment” will become the main way to build community-based prevention and control capacity for public health events.

Based on the “information-resources-subject-mechanism” analytical framework of community-based prevention and control capacity of public health events, the data-driven model is mainly reflected in the “technical empowerment” of community-based prevention and control of public health events from four aspects: data integration, process optimization, multi-level collaboration and smart operation.

First, the data integration of the information platform. The integration of massive decentralized and fragmented data is the basis for community-based prevention and control of public health events. In the prevention and control of public health events, the community-based public health prevention and control data integration should be realized through the construction of the information platform for information management, utilization and mining so as to improve data support capacity. First, based on the existing platforms of communities, governments and enterprises, a closed loop of prevention and control data of “access management-information mapping-event monitoring and management of key groups-information management and information application” was constructed. Through data mining of residents’ basic information and other information platforms, the flow situation of residents in the area is accurately identified with the development idea of “big data + grid”, which improves the efficiency of information collection and effectively reduces the statistical burden at the grass-roots level. At the same time, the refined management of “one person and one file” is implemented. Secondly, a data management system for the study and judgment of public health events should be constructed to realize efficient screening and monitoring of the development of public health events. Through the integration of dynamic public health event prevention and control data and information, such as household electricity consumption information and traffic information, precise prevention and control of key management objects under the jurisdiction can be carried out, emergency early warning work can be effectively deployed, and development space for emergency recovery can be left. Finally, through the data integration and in-depth mining of the information platform, the evaluation of different prevention and control situations and community-based prevention and control capacity is established, and the risk factors of different levels of the community are divided to realize the dynamic response and sustainable management of community-based prevention and control of public health events.

Second, the dredging of the resource allocation system. The dredging of the resource allocation system for community-based prevention and control of public health events mainly includes two levels: within the community and between communities. On the one hand, the dredging of prevention and control resource system within the community mainly includes prevention and control management resource system and medical treatment resource system. First, the dredging of the community-based prevention and control management resource system is mainly through the intelligent outbound call platform to efficiently check the key personnel in the jurisdiction; establish public health event prevention and control platforms, such as “On Call”; to grasp the dynamic health status of residents in real time; meet the individual needs of residents and liberate the community workers from the shackles of exhausting and tedious material statistics. Second, the dredging of the community medical treatment resource system mainly focuses on the four links of “screening-transportation-admission-treatment” and establishes an information sharing platform between community medical institutions and designated medical institutions so as to form a closed loop of initial diagnosis, referral, and delivery by community medical institutions and diagnosis and isolation treatment by designated medical institutions. Through the establishment of the “cloud clinic”, “public health medical observer management platform”, and service alliances between treatment hospitals and community hospitals, the medical treatment and protection channels of “susceptible groups-key groups-confirmed groups” are dredged. On the other hand, the dredging of the resource allocation system between communities is mainly to investigate the resource links and resource demand characteristics between communities based on the assessment of the community coping capacity, so as to build a regional community of community-based public health prevention and control.

Third, the collaborative structure of multiple subjects. The community-based diversified prevention and control functions of public health events determine the necessity of forming an effective governance system for prevention and control work. In order to perform the multiple functions of prevention and control management, the basic experience of community-based prevention and control is to build a diversified co-governance structure of cooperation inside and outside the community through resource expansion. On the one hand, in terms of stimulating the endogenous main body of the community, the awareness of daily management participation and the emergency management response of community residents should be effectively enhanced. In the prevention and control of the global infectious diseases that broke out in 2020, the “non-drug intervention measures” adopted by the Chinese government under strong government mobilization were recognized by the World Health Organization as a model of prevention and control in the history of world health, which is highly related to the strengthening of residents’ awareness of emergency management during the emergency period. In the daily management of residents, the awareness of emergency management should be guided, and a multi-level transmission system for daily management of a four-level prevention and control structure of “party member-unit-building-community” should be constructed on the basis of a comprehensive investigation of community residents. On the other hand, in terms of social and non-governmental resource links, a diversified collaborative structure of external community assistance can be constructed by expanding social power and volunteer resources in a data-driven model. It is also possible to reasonably guide multiple subjects to participate in public health prevention and control work and form a personnel organization system of joint prevention and control and mass prevention and control by dispatching college student volunteers, teachers and other party members from enterprises and institutions within the jurisdiction.

Fourth, the operation mechanism of the intelligent network. Risk society has a high degree of complexity and uncertainty, and the requirements for the performance of community prevention and control management functions are constantly increasing, which requires efforts to promote community smart governance with coordinated and unified leadership, interconnected and shared resources, multi-network collaboration, differentiated demand satisfaction and controllable association systems [45]. Building smart communities through information sharing and building community smart service solutions based on the full cycle of public health event development and multiple scenarios of social management are innovative ways for many communities to enhance their prevention and control capacity, including building network platforms and micro-community services to achieve digital technology empowerment and efficiency. For example, Jiangxi Xiangtang cooperates with the Headquarters of China Unicom to build the province’s first town-level big data platform for both peacetime and wartime use—“Smart Xiangtang”. Through inventory management, positioning management, node management and performance management, combined with intelligent terminal, big data technology to get through the whole process of administrative law enforcement and emergency management, it can realize the real-time monitoring and supervision of the whole process of the development of various affairs in the jurisdiction before, during and after the event.

## 6. Discussion

At the theoretical level, this study explores the generation process and internal mechanism of community-based prevention and control capacity of public health events from a new research perspective of governance element optimization.

First, different community-based prevention and control capacities generated by different governance models of public health events are explored based on different stages of prevention and control field. This study takes the optimization of governance elements of “information-resources-subject-mechanism” as the key generation condition for the construction of prevention and control capacity and proposes a theoretical analysis framework for the construction of community-based prevention and control capacity for public health events based on the optimization of governance elements. In the evolution of the governance model, from bureaucratic coordination to the data-driven model, various governance elements have been optimally allocated, forming a “circle-layer promotion effect” for community-based prevention and control of public health events. The “community inner circle” coordinated by the bureaucracy mainly improves the community-based prevention and control capacity of public health events through the mobilization of all internal subjects, such as party members and cadres, community workers, property companies, and neighborhood committees. When there is a “capacity shortcoming” in community prevention and control of public health events, data-driven efforts can be made to integrate information and resources inside and outside the community, mobilize social forces outside the community and mobilize volunteers to participate extensively so as to improve the prevention and control capacity of public health events through intelligent operation mechanisms. In this way, the “circle-layer promotion effect” of community-based prevention and control of public health events can be brought into full play through the linkage of governance bodies at the inner and outer layers so as to build a capacity system of prevention and control of public health events for both peacetime and wartime use.

Further, the data-driven community-based prevention and control capacity of public health events explores a digital organization and coordination model that is different from the bureaucratic coordination and governance model (see Table 2). Among them, the bureaucratic coordination model has formed an administrative order-based community prevention and control capacity for public health events. Taking administrative power and administrative orders as the basis for legitimacy, this capacity achieves optimal allocation of governance elements through top-down political mobilization and can play a good role in emergency management in the early stage of public health events. However, with the extension of community-based prevention and control process of public health events, it will face the “counter-function of bureaucracy”, such as high cost of prevention and control, insufficient manpower and material resources, rigid rules and instructions. Different from the bureaucratic coordination model, the data-driven model has formed a data-driven community-based prevention and control capacity for public health events, which takes information sharing and data interaction as the basis for legitimacy. In addition, this capacity realizes the optimal allocation of governance elements through horizontal data interaction and shared utilization of multiple governance subjects, thus greatly reducing governance costs, promoting resource sharing, and improving the ability of early warning, disposal and recovery of public health events.

Secondly, from the perspective of the internal mechanism of the formation of data-driven community-based public health prevention and control capacity, a new idea of data-driven embedded bureaucratic coordination to improve governance capacity is proposed. The advocates of the new public management theory have deeply analyzed the various drawbacks of rational bureaucracy, and proposed five strategies to abandon the core, consequence, customer, control, and culture of the bureaucracy [46], or advocated breaking through the bureaucracy system by building a service organization driven by users and creating new governance models such as market-based government, participatory government, flexible government, and regulation-dismiss government [47]. As shown in Table 2, although there are differences between bureaucratic coordination and a data-driven model in multiple dimensions, they are not a simple substitution relationship, but an embedded promotion relationship. The data-driven model does not mean a complete abandonment of the bureaucratic coordination model. Rather than dismissing rational bureaucracy, the study advocates an “embedded” view. On the one hand, in the early stage of community-based public health event prevention and control, the bureaucratic coordination model can quickly contain the spread of the event, which fully reflects the institutional advantage of “concentrating efforts on major events”. On the other hand, the data-driven model is not completely separated from the bureaucratic coordination model, but a new governance model that promotes the optimization of governance elements through the data-driven model on the basis of the bureaucratic coordination model. The combination of the data-driven model and executive order-based community public health prevention and control capacity reflects the adaptation of advanced technologies and systems.

## 7. Conclusions

**Figure 3:**
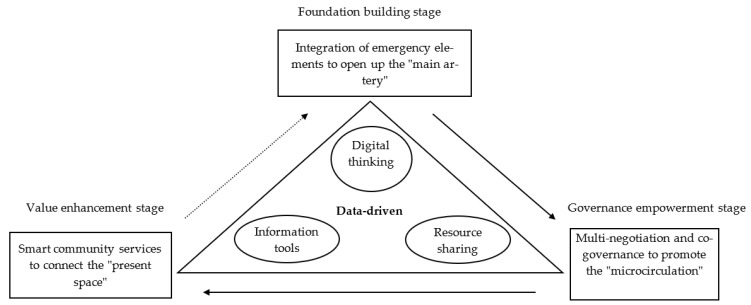
Three-step improvement path of community-based public health prevention and control capacity. Source: self-made.

The path to improve the data-driven community prevention and control capacity of public health events is the foothold of the study. The transition from bureaucratic coordination to a data-driven prevention and control model requires the transformation of digital thinking, the sharing of multi-party resources and the use of information tools. Through the data-driven model, the benign interaction between government management, social governance and residents’ self-governance under the community prevention and control of public health events can be realized. The building of data-driven community-based prevention and control capacity of public health events needs to seek breakthroughs in three stages: foundation building, governance empowerment and value enhancement (See Figure 3).

First, digital thinking is used to break through the “main artery” and improve the integration ability of emergency elements. The foundation construction is the first stage to drive the improvement of community-based prevention and control capacity of public health events. It is mainly to transform the management needs of community-based prevention and control of public health events into standardized risk identification and process control capacity by cultivating the management concept of digital governance. First, improve data integration capacity with information platform. On the one hand, through the integration of online and offline dual data and information, a basic information sharing platform for population management database identification is established, and offline prevention and control work data, such as community workers and prevention and control workers, are entered, and the resident information is modularized and decomposed. In this way, diverse and large-scale structured information on residents’ characteristics and unstructured daily demand preference data can be obtained online. On the other hand, through the flow of prevention and control information and the coordination of prevention and control resources, the “main artery” of community-based prevention and control is opened, and the data chain of integrated emergency elements is constructed to realize the dynamic management of the process structure of public health event prevention and control and form the full-data community-based prevention and control patterns of public health events with efficient information transfer and collaborative process operation by taking the resident data-driven model as the core. Secondly, digital coordination of resources is used to improve orderly management ability. Through digital coordination, such as information notification, allocation of prevention and control materials, and medical treatment channels, orderly management of human, financial and material resources in community prevention and control of public health events can be realized. The virtual flattening reform of the organization structure of public health events is promoted through information construction to realize the transformation from the current bureaucratic coordination model relying on internal and partial information of the administrative system to the data-driven pattern of co-construction, co-governance and sharing supported by upper and lower linkage information.

Second, resource sharing is used to promote the “microcirculation” and enhance the capacity for multi-negotiation and co-governance. Governance empowerment is the second stage of improving the data-driven prevention and control capacity of public health events, which will construct the main body structure of hierarchical negotiation and multiple governance dominated by the community, with the cooperation of government agencies, enterprises, public institutions and social organizations within the jurisdiction and the participation of community residents. The key point is to change from a centralized strategic positioning to a multi-participation emergency management strategy under contextualization. On the one hand, we need to fully tap endogenous resources in communities and enhance the core prevention and control capacity of diverse entities within communities. It is necessary to give full play to the advantages of community management, establish social capital network among community members and promote the sharing of resources among local authorities, enterprises and institutions, and social organizations. The microcirculation of unimpeded resources inside and outside the community will be opened up through the establishment of a four-level joint prevention and control organization structure of “community party committees–combination of grid members and secondary party organizations–party groups of each building–party member demonstration post”, and the capacity of negotiation and co-governance of multiple subjects will be improved. On the other hand, it is necessary to effectively stimulate social and non-governmental forces to enhance the collaborative prevention and control capacity of multiple external entities outside the community, give full play to the flexibility, efficiency and precision of non-governmental forces to build a guiding mechanism for social forces to participate in community prevention and control of public health events and realize the effective connection and integration of prevention and control resources of social forces and internal prevention and control resources of the community.

Third, information tools are used to connect the “present space” and enhance the community’s smart service capacity. Value enhancement is the third stage of improving the data-driven community-based prevention and control capacity of public health events. It mainly uses information tools to connect the “present space” of community residents’ needs and connects the deep integration of administrative forces at vertical levels and the network collaboration of external organizations at horizontal levels through big data, artificial intelligence and block chain technology so as to realize the linkage of each node in the community prevention and control of public health events and link into a common economic and social network and construct a community intelligent service model. In the specific operation process, Internet media, such as QQ groups, WeChat groups, and mailboxes, can be used to release information resources and expand the existing living space in combination with the needs of community users and public services. It can encourage the development of O2O community management model, actively connect vegetable and fruit production bases around the jurisdiction into the community and serve the community by introducing or developing profit or non-profit third-party organizations, such as vegetable and fruit e-commerce platforms, property management companies and voluntary organizations so as to realize the instrumental improvement of the way of interaction between community and residents and the sense of “good living” for all members. In addition, community online shopping malls can be built to gather community public resources to meet the diverse needs of residents, help groups with special difficulties and reduce the group vulnerability of special people in community-based prevention and control of public health events through the management of “ordering” by residents, “ordering” by communities and “sending orders” by volunteers.

## Figures and Tables

**Table 1:** Example of open coding for community-based public health prevention and control.

Coding	Content Representation	Conceptualization	Category
SZD01	Lack of community-based prevention and control platform planning and cross-border technical cooperation, lack of business extension and connection with community hospitals, transportation, civil affairs, communications and other platforms, lack of full data flow information for the entire process of epidemic prevention and control under population mobility.	Full data flow information missing	Data Segmentation for Information Transfer
SBJ02	The allocation of prevention and control resources, such as people and property in the community and epidemic statistics, are still mainly based on inefficient manual records and manual verification, and the retention rate and update rate of the ledger are low.	Inefficient manual recording	
QSM01	The construction of the information platform could not be realized in the short term. They adopted the most primitive method—huge-crowd strategy, organized a volunteer team of 100 people, divided labor and cooperated, and called 61 designated hospitals, 89 community health service centers and 549 medical institutions, collected and released the data of all medical institutions in the province every day.	Missing information platform	
SXA03	Some patients are reluctant to go to the isolation point, hide at home to avoid investigation, and I often knock on the door for a long time without answering and had to look at the meter. If the meter was running, it proved that there was someone at home, and then I would knock on the door again.	Lack of deep integration and association recombination	
SBF04	The 60 staff of the entire community health service center only have one set of protective clothing, which is repeatedly used with ultraviolet radiation. And who receive suspected patients who will wear it.	Lack of medical resources	Coordination Obstruction of Emergency Resources
ZZY01	Within 10 days, documents were sent from the Municipal Party Committee and Municipal Government, the Organization Department of the Municipal Party Committee, the District Government, and the Public Security Bureau. Every day, the pressure on the grassroots level was not small.	Multiple prevention and control functions	
SHS05	Due to limited inspection equipment and personnel allocation, community hospitals are faced with operational standard problems, such as sample transfer, detection and disposal, in the actual operation process, which put forward practical challenges to their own biosafety protection.	Lack of medical resources	
SJQ06	The management staff of the community service center were quarantined for 14 days because they had visited confirmed cases, leaving only three social workers to carry out heavy epidemic prevention work.	Lack of grassroots investigators and protection resources	
SJH07	For the first two days of the concentrated quarantine, there was no clear method of quarantine, no medical staff and no medicine, despite the presence of a quarantine site. As a result, complaints are again being snowed into the community.	Lack of grassroots investigators and protection resources	
SJH08	According to government documents, coercive measures can be taken against those who are unwilling to be quarantined, but only the community police can enforce the coercive measures. The community does not have this authority and can only connect to it, but it always takes time to connect.	Task allocation system	Loose Combination of Autonomous Systems
QSM02	There are abundant volunteer teams, including those from the Health commission, prevention and control headquarters, hospitals, medical equipment and disaster relief. Ultimately, he relies on the volunteers’ personal connections to get things done. However, he believes that this is not a sustainable process of operation, which completely relies on human resources to get through.	Unsustainable network resources	
QHB03	Although many volunteers and social organizations have also acted quickly in the collection of protective materials, most of these actions exist in loose organizations. Seeing that there is a need on the front line, they do it in a hurry, and the progress is very fast, but it is difficult to achieve real coordination and cooperation. In the end, online support did not form a community-based investigation pattern of close cooperation with the community offline.	Loose organization	
SQY09	Community hospitals do not have the most direct distribution advantage in the allocation of resources, such as manpower, capital, equipment, and materials. In emergency events, such as epidemic prevention and control, the deployment of emergency reserve materials and prevention and control personnel depends on hierarchical allocation.	Hierarchical diagnosis and treatment system faults	Mechanism Fault of Coordination and Linkage
QBN04	Action-oriented public welfare organizations and professional rescue teams were left behind. For them, unable to access the disaster site, and very helpless.	Lack of volunteer assistance	
SWN10	Basically, the community neighborhood committee is paralyzed, the community cannot solve the problem of seeking a doctor, and the patient can only bypass the community and go directly to the hospital.	Lack of direct reporting system of epidemic situation in grassroots communities	

According to the research paper specifications, S is used when the public health prevention and control implementation body is the community, Z is used when the implementation body is the province or city and Q is used when the implementation body is the enterprise unit, social organization, etc. The first letter of the name of the community, the place name of the province and the city and the name of the enterprise unit are used for the secondary coding and the last digit is the case number. Since the main source of the cited content of the case is the factual content in the reports of various authoritative websites, we will not specify the cited content one by one and would like to express our thanks to the cited authors.

**Table 2:** Comparison between bureaucratic coordinated prevention and control model and data-driven prevention and control model.

	Bureaucratic Coordinated Prevention and Control Model	Bureaucratic Coordinated Prevention and Control Model
Basis of legitimacy	Executive power	Information sharing
Governance model	Political mobilization, concentration of power, executive order	Network platform, data interaction, cooperation and co-governance
Content focus	Vertical mobilization of various resources, high efficiency and high cost	Horizontal coordination of various resources, high efficiency and low cost
Governance performance	Fast and efficient, high cost	Fast and efficient, resource sharing
Governance structure	Bureaucratic coordination	Horizontal collaboration
Governance applicability	Early stage of prevention and control	Middle and late stage of prevention and control
Capacity building	Executive order-based community public health prevention and control capacity	Data-driven community public health prevention and control capacity

Source: self-made.

## Data Availability

Not applicable.
